# Characterization of Off-Gases from an Inert Electrode Aluminum Electrolysis Cell

**DOI:** 10.1007/s40831-025-01348-0

**Published:** 2025-12-04

**Authors:** Samuel Senanu, Gudmundur Gunnarsson, Daniel Gunnarsson, Ole Kjos, Heiko Gaertner, Rauan Meirbekova, Jon Hjaltalin Magnusson

**Affiliations:** 1 SINTEF Industry, Trondheim, Norway; 2 IceTec, Reykjavik, Iceland; 3 Arctus, Reykjavik, Iceland

**Keywords:** Inert electrode, Electrolysis, Aluminum, Off gas, Characterization

## Abstract

Characterization of the off-gases from a 500 A inert electrode aluminum electrolysis cell operating at a temperature of ca. 800 °C has been conducted using characterization units comprising gas chromatography, tuneable diode laser spectroscopy, and Fourier-transform infrared spectroscopy. The off-gases detected from the inert electrode aluminum electrolysis cell have been compared to the off-gases released from the traditional Hall–Héroult aluminum electrolysis cell. All the measurements from the inert electrode cell confirmed oxygen as the main process gas during the electrolysis process. Hydrogen fluoride, HF, which is assumed to result from reactions between moisture in the alumina raw material or the surrounding air and the fluoride melt, was also observed together with gases assumed to come from air introduced, such as nitrogen and CO_2_. There were no indications that these gases came from the production process. A very small amount of SiF_4_ was also detected.

**Graphical Abstract:**

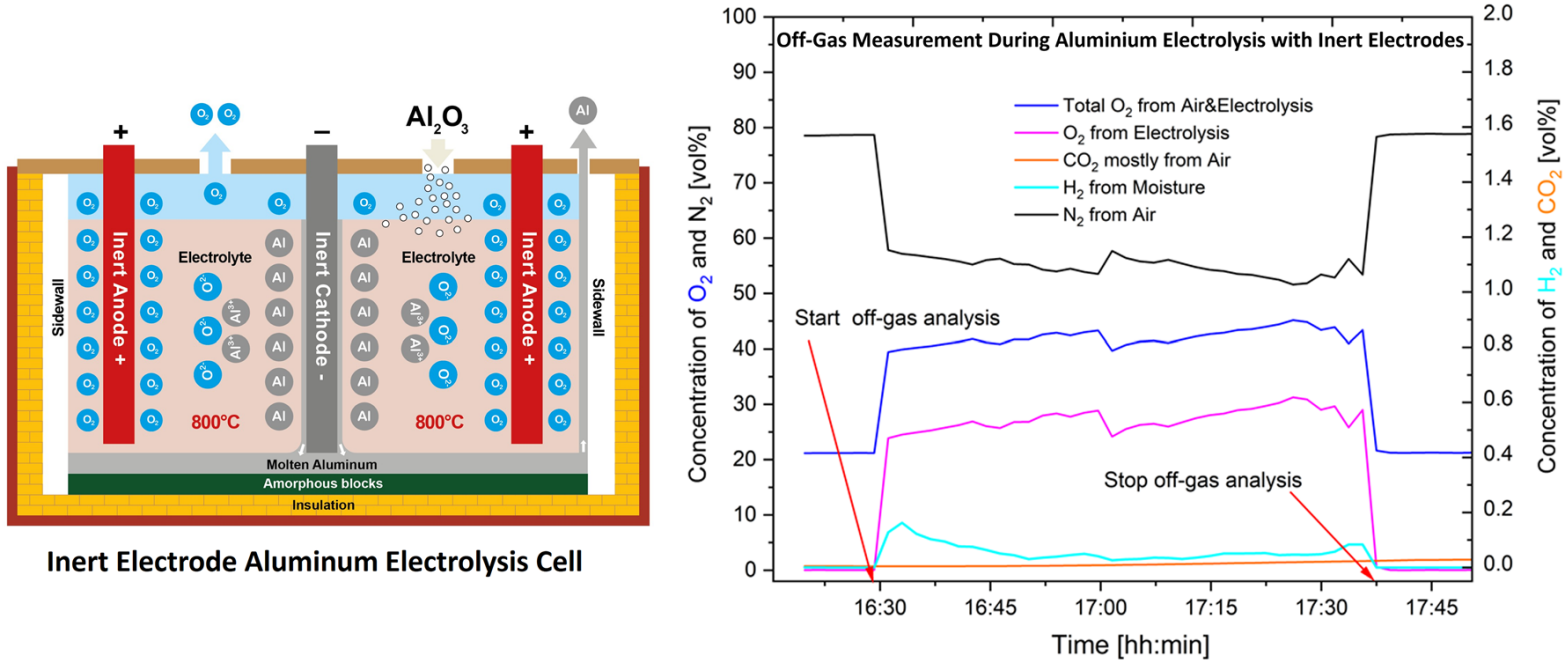

## Introduction

### The Aluminum Electrolysis Process

#### Hall–Héroult Process and Off-Gas Formations

**Fig. 1:**
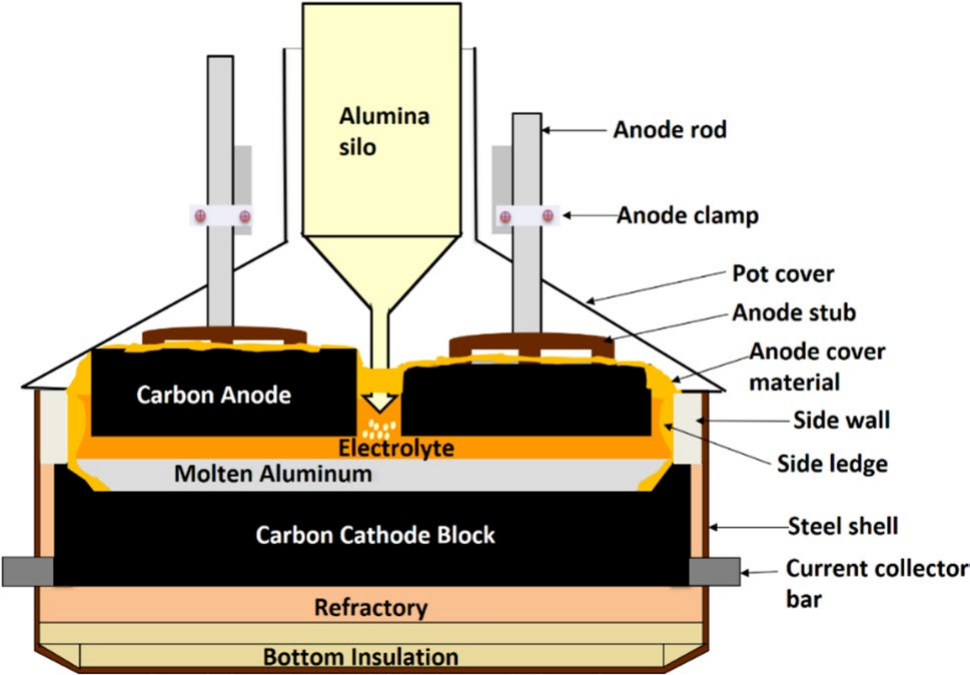
Cross-sectional sketch of the Hall–Héroult electrolysis cell based on prebaked technology

The conventional process to produce aluminum metal is the Hall–Héroult process. This process involves the electrolytic reduction of aluminum oxide, Al_2_O_3_, dissolved in a fluoride melt consisting mainly of cryolite, Na_3_AlF_6_, using carbon as the anode material in an electrochemical reduction cell [1, 2]. Two electrochemical reduction cell designs or technologies based on the carbon anode exist for the Hall–Héroult process in the aluminum industry. These two anode designs are the prebaked anodes and the Søderberg anodes. The prebaked anode design, which involves baking the carbon anodes before inserting them into the electrochemical reduction cell, is the dominant one for the industry. The Søderberg anodes design involves the in-situ baking of the carbon anodes using the waste heat from the process [1, 2]. The carbon anode is consumed during the production process and needs replacement either as baked anodes, as in the case of the prebaked design, or anode paste briquettes, as in the case of the Søderberg design [1, 2]. The temperature of the molten cryolite electrolyte used in the Hall–Héroult process is typically kept between 955 to 965 °C for optimal operations in most modern smelters. A cross-sectional sketch of the electrochemical reduction cell based on the prebaked anode design is given in Fig. 1.

The overall reaction for the Hall–Héroult process is given by Eq. (1).

1 $$ \frac{1}{2}{\mathrm{Al}}_{2} {\mathrm{O}}_{3} \left( {{\mathrm{diss}}} \right)\, + \;\frac{3}{4}{\mathrm{C}}\left( {\mathrm{s}} \right) \; \to \;{\mathrm{Al}}\left( {\mathrm{l}} \right)\; + \;\frac{3}{4}{\mathrm{CO}}_{2} \left( {\mathrm{g}} \right) $$

The off-gases released during the Hall–Héroult electrolysis process are mostly related to the carbon anodes, the fluoride melt and moisture from the aluminum oxide raw material and the environment. The off-gases from the process contain potent greenhouse gases like CO_2_ and perfluorocarbons such as CF_4_ and C_2_F_6,_ as well as other gases, such as HF, SO_2_, COS, CO, etc. [3]. The section below will elaborate on the formation of some of these off-gases during the aluminum production process via the Hall–Héroult process, see Eqs. (2)–(4).

2 $$ {\mathrm{C}}\; + \;{\mathrm{O}}_{2} \; = \;{\mathrm{CO}}_{2} $$

3 $$ {\mathrm{Al}}\left( {{\mathrm{diss}}} \right)\; + \;\frac{3}{2}{\mathrm{CO}}_{2} \; = \;\frac{1}{2}{\mathrm{Al}}_{2} {\mathrm{O}}_{3} \left( {{\mathrm{diss}}} \right)\; + \;\frac{3}{2}{\mathrm{CO}} $$

4 $$ {\mathrm{C}}\; + \;{\mathrm{CO}}_{2} \; = \;2{\mathrm{CO}} $$

The sulphur content within the carbon anodes, due to the use of petrol cokes for their production, contributes to the formation of sulphurous gas species. Carbonyl sulphide, COS, is the primary product, while sulphur dioxide, SO_2_, is formed by oxidation, see Eqs. (5)–(8).

5 $$ \frac{1}{2}{\mathrm{Al}}_{2} {\mathrm{O}}_{3} \left( {{\mathrm{diss}}} \right)\; + \;\frac{3}{2}{\mathrm{C}}\; + \;\frac{3}{2}{\mathrm{S}}\; = \;{\mathrm{Al}}\left( {\mathrm{l}} \right)\; + \;\frac{3}{2}{\mathrm{COS}} $$

6 $$ {\mathrm{COS}}\; + \;\frac{3}{2}{\mathrm{O}}_{2} \; = \;{\mathrm{SO}}_{2} \; + \;{\mathrm{CO}}_{2} $$

7 $$ {\mathrm{COS}}\; + \;2{\mathrm{CO}}_{2} \; = \;{\mathrm{SO}}_{2} \; + \; 3{\mathrm{CO}} $$

8 $$ {\mathrm{SO}}_{2} \; + \;\frac{1}{2}{\mathrm{O}}_{2} \; = \;{\mathrm{SO}}_{3} $$

The fluoride electrolyte used in the production process results in the generation of hydrogen fluoride gases owing to the reaction between the fluoride components and moisture, as illustrated in Eqs. (9) and (10).

9 $$ {\mathrm{H}}_{2} {\mathrm{O}}\; + \;\frac{2}{3}{\mathrm{AlF}}_{3} \left( {{\mathrm{diss}}} \right)\; = \;\frac{1}{3}{\mathrm{Al}}_{2} {\mathrm{O}}_{3} \left( {{\mathrm{diss}}} \right)\; + \;2{\mathrm{HF}}\left( {\mathrm{g}} \right) $$

10 $$ {\mathrm{H}}_{2} {\mathrm{O}}\left( {\mathrm{g}} \right)\; + \;\frac{2}{3}{\mathrm{NaAlF}}_{4} \left( {\mathrm{g}} \right)\; = \;2{\mathrm{HF}}\left( {\mathrm{g}} \right)\; + \;\frac{2}{3}{\mathrm{NaF}}\left( {{\mathrm{diss}}} \right)\; + \;\frac{1}{3}{\mathrm{Al}}_{2} {\mathrm{O}}_{3} \left( {{\mathrm{diss}}} \right) $$

Aside the formation of HF, which results from fluoride components, the presence of carbon due to the carbon anode in a system consisting of fluoride components leads to the formation of perfluorocarbon gases, namely, tetrafluoromethane, CF_4_ and hexafluoroethane, C_2_F_6_. These gases are formed when the oxide content in the electrolyte is depleted leading to the decomposition of the fluoride electrolyte. Perfluorocarbon gases are greenhouse gases with higher global warming potential than CO_2_. The reactions for their formation are given in Eqs. (11) and (12).

11 $$ {\mathrm{Na}}_{3} {\mathrm{AlF}}_{6} \; + \;{\mathrm{C}}\; = \;\frac{3}{4}{\mathrm{CF}}_{4} \; + \;{\mathrm{Al}}\left( {\mathrm{l}} \right)\; + \;3{\mathrm{NaF}} $$

12 $$ {\mathrm{Na}}_{3} {\mathrm{AlF}}_{6} \; + \;{\mathrm{C}}\; = \;\frac{1}{2}{\mathrm{C}}_{2} {\mathrm{F}}_{6} \; + \;{\mathrm{Al}}\left( {\mathrm{l}} \right)\; + \;3{\mathrm{NaF}} $$

## Inert Electrode Process and REVEAL Project

**Fig. 2:**
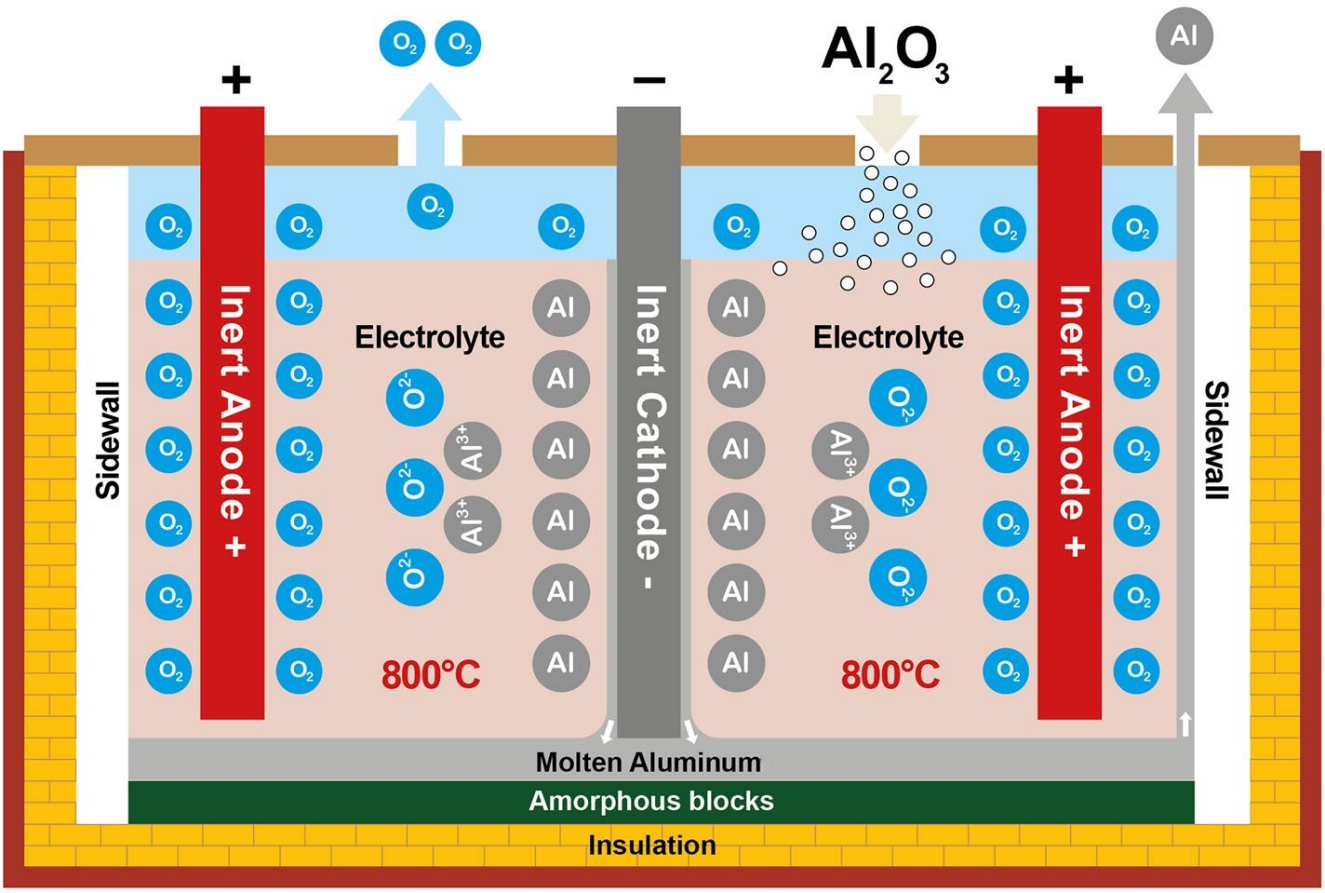
Vertical inert electrode technology setup used for the REVEAL project [4]

The Revolutionary energy storage cycle with carbon-free aluminum (REVEAL) project is a Horizon EU project aimed at producing carbon-free aluminum for seasonal energy storage using inert electrodes. The inert electrodes proposed by the REVEAL project are arranged vertically and consist of metallic inert anodes composed of Ni–Fe–Cu-based alloys and TiB_2_ cathode material [4–6], see Fig. 2. This eliminates the formation of any carbon-containing gases due to the absence of carbon anodes. This implies that gases such as perfluorocarbons, CO_2_, CO, COS, SO_2_ and SO_3_ that are related to the carbon anodes are not expected for the inert electrode process. The main reaction gases that form from the process are O_2_ and HF. HF is formed due to the fluoride components reacting with moisture, as in the case of the Hall–Héroult process. Oxygen gas is the main gaseous reaction product for the aluminum production process, as illustrated in Eq. 13.

13 $$ \frac{1}{2}{\mathrm{Al}}_{2} {\mathrm{O}}_{3} \left( {{\mathrm{diss}}} \right)\; = \;{\mathrm{Al}}\left( {\mathrm{l}} \right)\; + \;\frac{3}{4}{\mathrm{O}}_{2} $$

Aside from the environmental nature of the inert process, the temperature of the electrolyte employed is ca. 800 °C, which is more than 150 °C lower than the Hall–Héroult process at ca. 955 to 965 °C [4].

The low temperature electrolyte can be described as mixture of sodium cryolite ((NaF)_3_AlF_3_), potassium cryolite ((KF)_3_AlF_3_), and aluminum fluoride (AlF_3_), with the composition being defined by the molar ratios CR and KR, as given in Eqs. (14) and (15).

14 $$ {\mathrm{CR}}\; = \; \frac{{\left[ {{\mathrm{NaF}}} \right]\; + \;\left[ {{\mathrm{KF}}} \right]}}{{\left[ {{\mathrm{AlF}}_{3} } \right]}} $$

15 $$ {\mathrm{KR}}\; = \; \frac{{\left[ {{\mathrm{KF}}} \right]}}{{\left[ {{\mathrm{NaF}}} \right]\; + \;\left[ {{\mathrm{KF}}} \right]}} $$

In the experiments described here the CR ratio was 1.30 and KR ratio was 0.80 in the April experiment and 0.64 in the November experiment. For comparison it can be mentioned that the electrolyte used in in the Hall–Héroult process rarely contain any potassium fluoride (KR = 0) and that the CR ratio is about 2.20, showing that the electrolyte in the inert electrode process contains much more AlF_3_ than the Hall–Héroult electrolyte. The increased content of content of AlF_3_ is crucial in lowering the electrolyte temperature but reduces alumina solubility. The reduction in alumina solubility with increased AlF_3_ is partly counteracted by increasing the KR ratio. Alumina solubility at 800 °C in electrolyte with CR = 1.30 and KR = 0.80 is estimated to be 5.7 wt%, and for CR = 1.30 and KR = 0.64, alumina solubility is estimated to be 5.0 wt%, based on literature data [7]. The relatively low temperature of the inert electrode technology helps to reduce the consumption of the anode material.

### Off Gas Characterization in the Aluminum Industry

Several techniques exist for the characterization of off-gases from the aluminum electrolysis process. Techniques such as Fourier-Transform Infrared Spectroscopy (FTIR), Tuneable Diode Laser Spectroscopy (TDLAS), Gas Chromatography (GC), Mass Spectrometry (MS), Differential Optical Absorption Spectroscopy (DOAS), etc., exist in the industry [3]. Each of these techniques has its advantages and strengths; however, the FTIR, GC and TDLAS techniques have been chosen for the gas measurement and characterization techniques planned for the REVEAL project. The following is a short overview of these techniques.

#### Fourier-Transform Infrared Spectroscopy

FTIR spectroscopy is a well-known technique for off-gas measurements and characterization in industry and involves the application of a polychromatic light source over the mid-infrared range. An advantage of FTIR is its versatile nature, allowing it to cover many relevant analytes. Data gathered during the measurements are converted from time to frequency domain using a Michelson interferometer. Employing multivariate regression, such as partial least squares (PLS), robust calibration models can be established and used for the interpretation of measurement results. Establishing a robust calibration model is very important to limit interference from species such as water, CO_2_ and SO_2_ during measurements. Despite the versatile nature of FTIR, it cannot be employed for homonuclear species, since they do not provide absorption in the mid-infrared range. Thus, another technique has to be considered when measuring and characterising homonuclear gas species, such as O_2_, H_2_, N_2_, etc. [8].

#### Gas Chromatography

Gas chromatography is an analytical technique employed to separate and detect the chemical composition of a sample mixture that can be vapourised without decomposing. It involves the application of chromatographic separation of gas analytes through a column and using different types of detectors to determine the quality and quantity of the gas present. Commonly used detectors include flame ionisation detector (FID), thermal conductivity detector (TCD), etc. Limitations of the detectors are related to the sensitivity to the analyte and their stability [3].

#### Tuneable Diode Laser Spectroscopy

This is an infrared absorption technique that utilises a diode laser as a narrow-bandwidth light source and operates in the near-infrared range (NIR, 700–2500 nm) and mid-infrared range (MIR, 2500–25000 nm). The sensitivity of TDLAS depends on analyte-specific absorptivity and light path length. The instruments are commonly operated in two sampling configurations, namely, extractive and in-line. Extractive sampling is often used for multi-phase analysis, where a measuring cell is used to increase path length. High precision mirrors guide the laser beam several times through the sample volume (measuring cell) thereby obtain the desired sensitivity. However, the trade-off for sensitivity is a loss in dynamics owing to the increased volume of the measurement cell to be filled with representative sample gas.

For in-line configurations, the quantification limit is dictated by the across-duct installation. The implementation of TDLAS is normally limited to single analytes; however, it is possible to tune the diodes to cover more analytes [3].

## Experiment

### Inert Electrode Aluminum Electrolysis Cell Setup

**Fig. 3:**
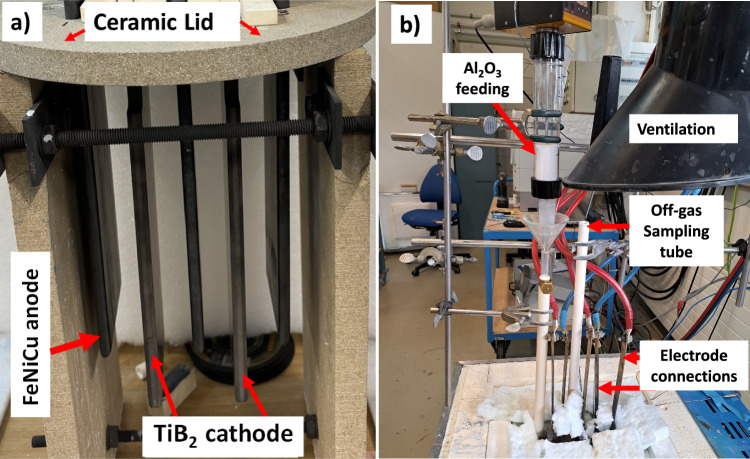
Experimental setup: **a** inert electrode setup fitted onto the ceramic lid, **b** full electrolysis cell setup

Two sets of electrolysis experiments were conducted in April and November of 2024 to complete the off-gas measurements due to transportation issues with the characterization units. The inert electrode setup to produce carbon-free aluminum for the two experiments involved vertically arranged inert electrodes made of FeNiCu alloy as the anode and TiB_2_ as the cathode. The vertically arranged inert electrodes were set up as a five-electrode system with two TiB_2_ cathodes and three FeNiCu anodes. The cathodes were sandwiched between the anodes in an anode–cathode–anode–cathode–anode (ACACA) configuration, see Fig. 3a. Furthermore, the alumina crucible used for the experiment in April had an internal diameter of 219 mm and an internal height of 321 mm, whereas the crucible used in November had an internal diameter of 250 mm and an internal height of 314 mm. An electric furnace was used for heating the crucibles during start-up and during the experiments. To avoid leakages of electrolyte to the furnace due to failure or excessive corrosion by the electrolyte, the alumina crucible in the April trial was placed in a stainless-steel crucible, with the annular space between the alumina and steel crucible filled file chamotte sand, which could absorb electrolyte in case of leakage. In the November experiment, the alumina crucible was placed in a graphite crucible, which was placed in a stainless-steel crucible to prevent oxidation of the graphite. The graphite crucible could hold the electrolyte in case the alumina crucible was breached, as well as evenly distribute heat to the alumina crucible.

The raw materials for electrolyte preparation were sodium cryolite and potassium cryolite from Solvay Fluor GmbH, aluminum fluoride from Alufluor AB and alumina from aluminum Oxid Stade GmbH. Solid unalloyed aluminum from Nordural was placed in the bottom of each crucible to give a metal pad of ca. 30 mm thickness. The electrolyte materials were dried overnight at 300 °C and then cooled to 100 °C, after which a known amount of the electrolyte, which corresponds to a CR = 1.30 and KR = 0.80 in the April experiment and to CR = 1.30 and KR = 0.64 in the November experiment, was mixed. The content of the crucible was then heated gradually to a temperature of 650 °C. The addition of electrolyte material was then done until the target electrolyte level was reached, after which the whole setup was heated to the operating temperature of ca. 800 °C.

A ceramic lid of 20 mm thick was specially designed to fit the electrodes and have openings for the gas measurement instruments and the alumina/electrolyte feeders. The electrodes were then fitted onto the ceramic lid and pre-heated to ca. 500 °C before inserting into the molten electrolyte at ca. 800 °C to avoid thermal shock damage. Figure 3a shows the electrode setup fitted onto the ceramic lid. The electrode-lid assembly was removed from the 500 °C furnace, and the electrodes were immediately inserted into the molten electrolyte in the alumina crucible. After insertion of electrodes, the ceramic lid rested on the alumina crucible. After insertion of the electrodes the electrolyte temperature dropped to about 730–740 °C, after which the electrolyte temperature was allowed to restabilise, with electrolysis starting at around 790 °C.

Feeding of smelter-grade alumina during the electrolysis was done via powder dosers from Lambda Instruments. The feeding rate was adjusted to correspond to the feed requirements corresponding to 80% current efficiency. The powder dosers and off-gas sampling tubes were connected to alumina tubes, which can be seen in Fig. 3b. To counteract evaporations changing the electrolyte composition, AlF_3_ was fed along with the alumina.

The cell amperage available for the electrolysis experiments was at ca. 400–600 A, depending on the anode current density and electrode dimensions. The current density was kept at ca. 0.8 A/cm^2^ for the April experiments and set to 0.7 A/cm^2^ for the November experiments. The current density for the present setup is calculated by assuming that half of the current goes to the central anode and that the current is evenly distributed on both sides of the central anode facing the two cathodes and neglecting any edge currents. This is called the nominal anode current density, or nominal CD.

### Off-Gas Characterization Setup

**Fig. 4:**
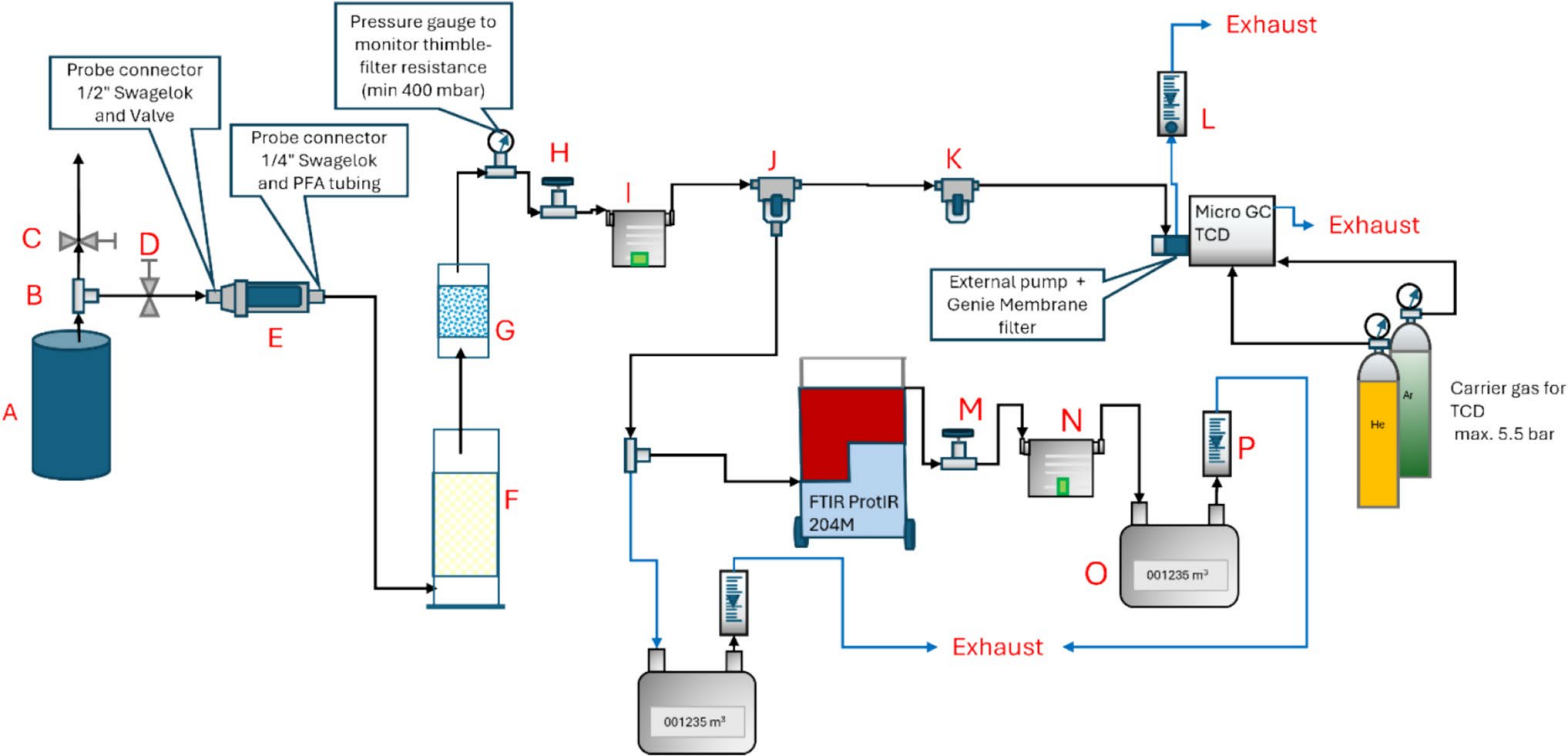
Off-gas sampling system: **A** electrolysis cell, **B** off-gas splitting unit, **C** valve for off-gas to ventilation system, **D** valve for off-gas sampling, **E** thimble filter, **F** HF scrubbing unit with activated alumina, **G** water vapour scrubbing unit with silica gel, **H**, **M** needle valve, **I**, **N** membrane pump, **J** sintered metal filter, **K** sintered metal filter, **L**, **P** flow meter, **O** diaphragm gas meter

Figure 4 shows the schematic setup of the off-gas sampling system that was planned for the off-gas characterization activity. However, as mentioned earlier in Section “Inert Electrode Aluminum Electrolysis Cell Setup” above, two measurement campaigns had to be organised as the FTIR equipment (FTIR ProtIR 204 M) was not available during the first campaign due to transportation issues.

Regarding the sampling of the off-gases, certain precautions had to be taken to safeguard the very sensitive and fragile equipment. As an example, to prevent electric short-circuiting, the sampling lines were connected to the extractor with a PFA tubing. The first activity involved splitting the off-gases coming from the top of the electrolysis cell at connection B indicated in Fig. 4. The majority of the off-gas was let off to the ventilation system via valve C, whereas the off-gas to be characterised was introduced at valve D. To avoid destruction and excessive exposure of the instruments, the sampled off-gas were first guided through different filters. The first filter was the thimble filter, E. This filter helped to reduce the condensates within the off-gas. HF was reduced using an activated alumina column, F, whereas water vapour was reduced in a scrubber, G, filled with silica gel. The main sample flow was guided through a flow meter, L, and left the sampling system through an exhaust tube venting the off-gas out via the ventilation system. Pressure conditions on both sides of the membrane pump were monitored with the help of a digital pressure gauge to detect filter blockage and ensure sufficient sample gas flow. The filter thimble, E, was changed as soon as the filter resistance caused the pressure on the low-pressure side of the membrane pump to drop below a certain value. An Agilent 490-PRO Micro-GC was used to monitor H_2_, O_2_, N_2_, CO_2_ and CO.

For the sampling during the November experiment, a different setup was used as the focus was less on identifying O_2_, and more shifted towards monitoring for unwanted trace components, and estimating HF levels. A combination of FTIR (Protea AtmosFIR) and TDLAS (NEO Laser Gas II HF) was, therefore, selected. The AtmosFIR cannot estimate O_2_ levels, a ZrO_2_ sensor was, therefore, used to estimate O_2_ levels, which unfortunately becomes pacified in HF environments. Some measurements were, therefore, done with active alumina to scrub the HF content to verify that O_2_ levels were on par with earlier measurements, while the main part of the analysis was conducted without active alumina or silica scrubbing of the gas.

## Results and Discussion

### Inert Electrode Aluminum Electrolysis Cell Operation

The critical parameters indicative of an aluminum electrolysis cell operation, such as cell voltage, electrolyte temperature and composition, alumina feeding and metal tapping, were monitored during the measurement campaigns in April and November and presented in the figures given in the following paragraphs.

**Fig. 5:**
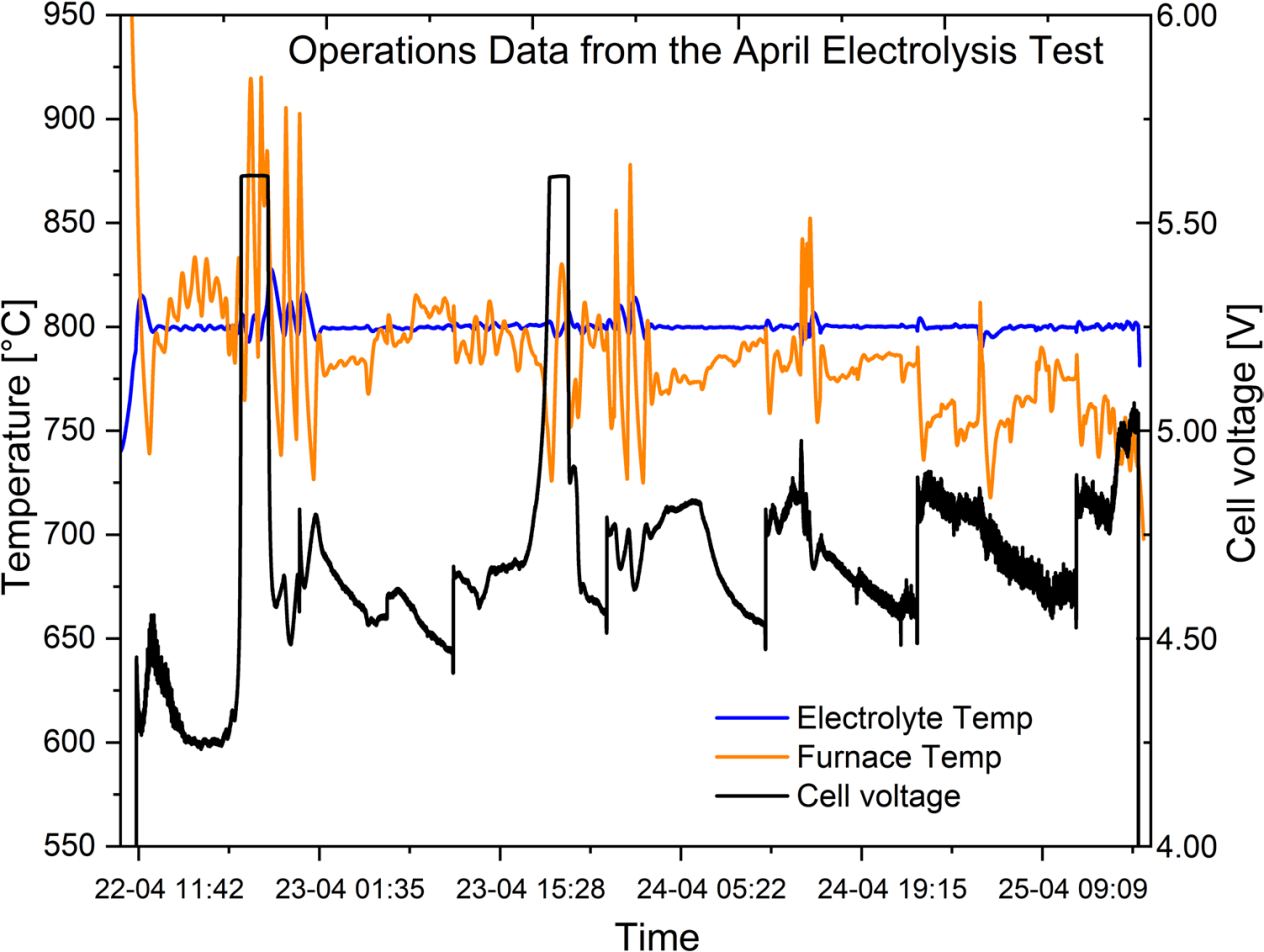
Cell voltage, electrolyte temperature and furnace temperature during the April 2024 electrolysis experiment with the REVEAL cell

**Fig. 6:**
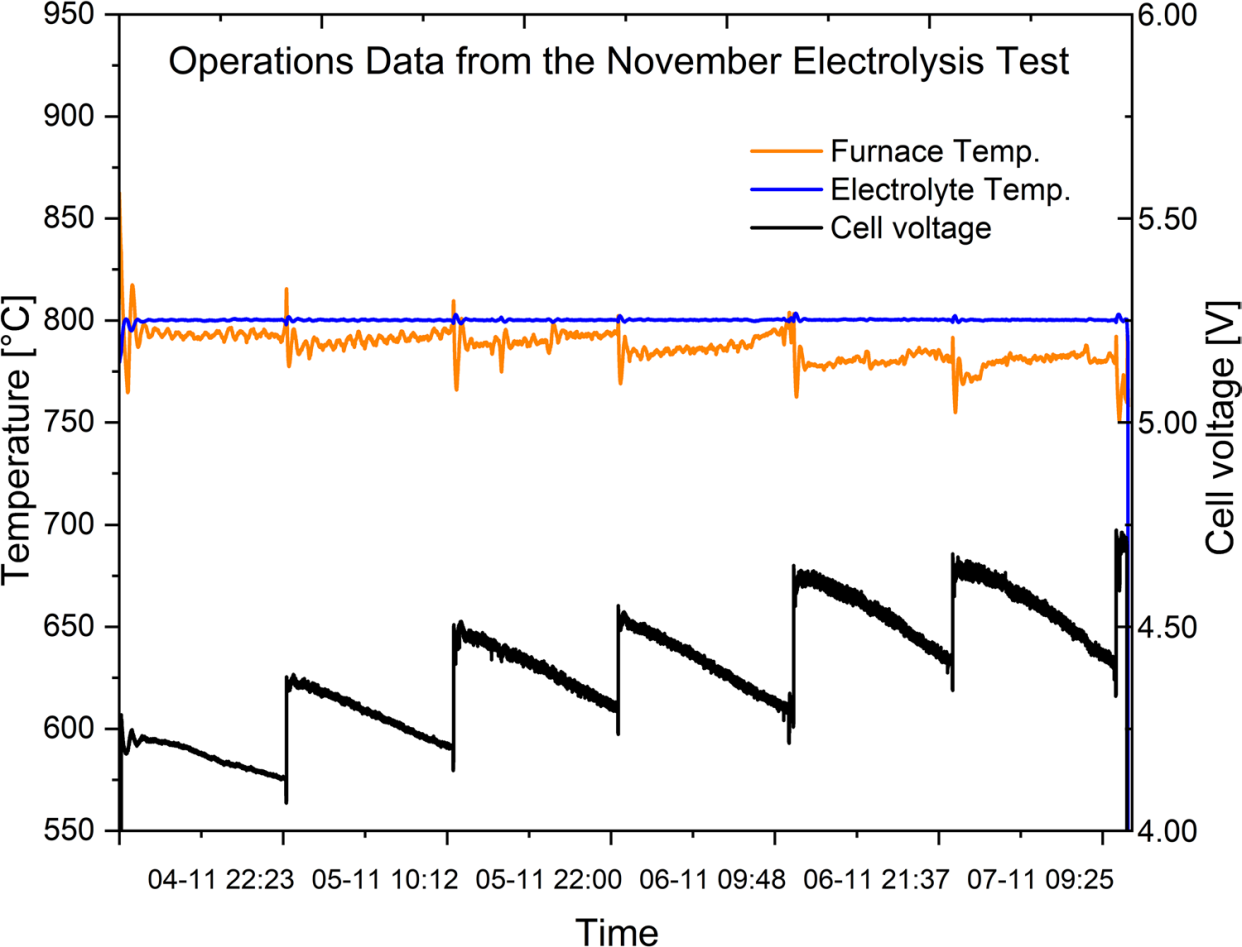
Cell voltage, electrolyte temperature and furnace temperature during the November 2024 electrolysis experiment with the REVEAL cell

During the April experiments, it was observed that the cell voltage increased to the set maximum voltage of 6 V during the first hours of the test. This was seen to result from excessive alumina feeding. After turning alumina feeding off for some time, the voltage went back to the normal expected value. Alumina feeding was then resumed, but on the second day of the experiment the voltage increased again to the set maximum voltage of 6 V. After turning off alumina feeding and making some adjustments to the alumina feeding rate, it was possible to avoid this sudden increase in voltage, see Fig. 5. Note, in Figs. 5 and 6, the voltage is presented as ‘Cell voltage’, where the voltage drops over the cables have been subtracted. The variations seen with the voltage after the initial instabilities have to do with the metal tapping operation. During the electrolysis process, the metal level increases due to production, and this leads to a gradual reduction in the voltage. Aluminum was tapped from the cell about every 12 h using a specially designed tapping system based on vacuum suction. During tapping of metal from the bottom of the crucible, the metal and the electrolyte level decreased suddenly, leading to a sudden increase in voltage, since the tapping only took about 5–15 s. The aluminum tapped in each tapping was from about 1100 g to about 1700 g. The level of electrolyte in the cell was measured during electrolyte sampling, and before and after tapping of aluminum from the bottom of the cell.

The experience from the April measurements regarding the alumina feeder setup made it possible to have a good start for the November experiments. The voltage was much more stable right from the start. This is given in Fig. 6.

### Off-Gas Characterization from Inert Electrode

**Fig. 7:**
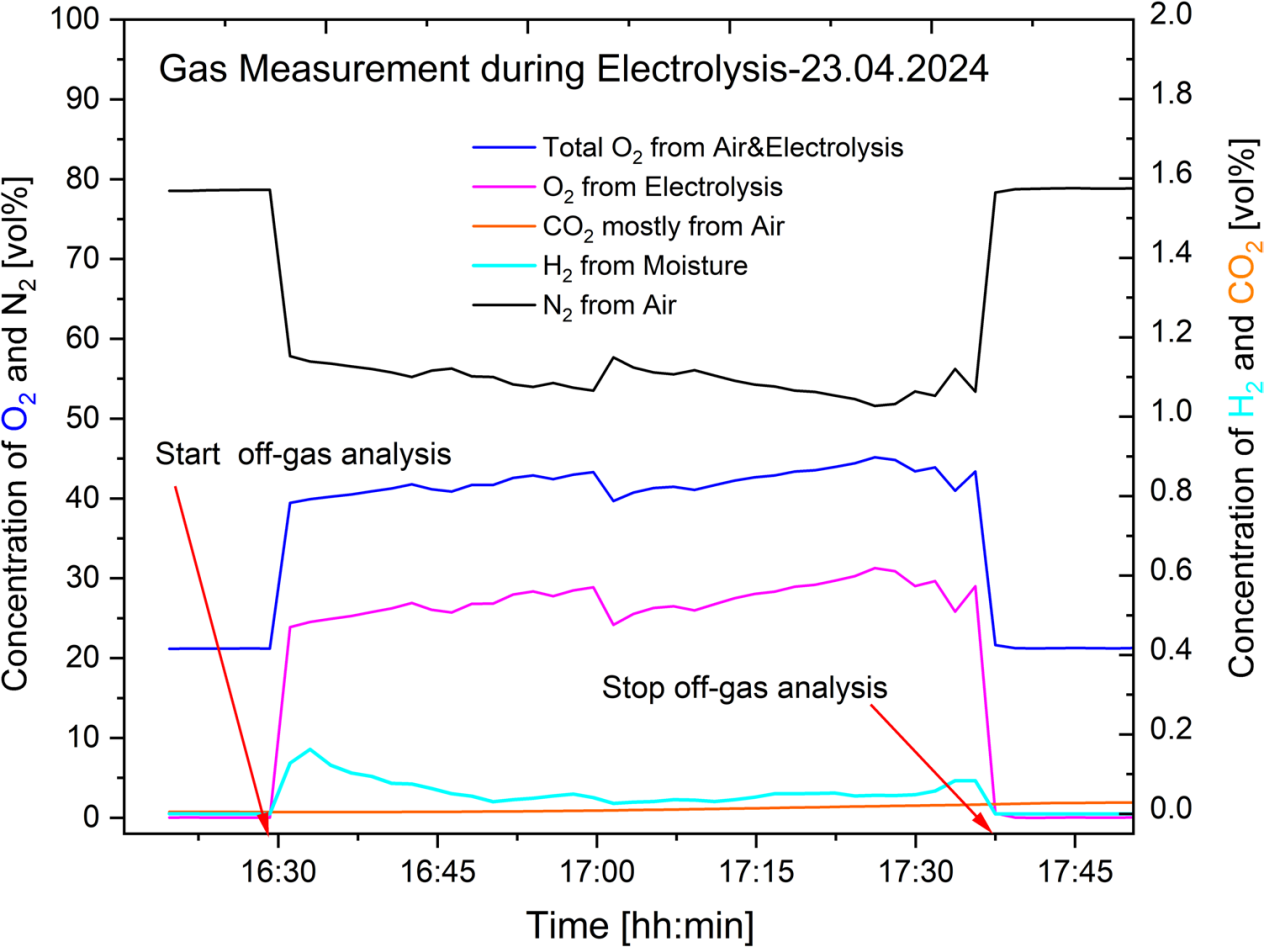
Off-gases from the REVEAL electrolysis process measured on 23.04.2024

**Fig. 8:**
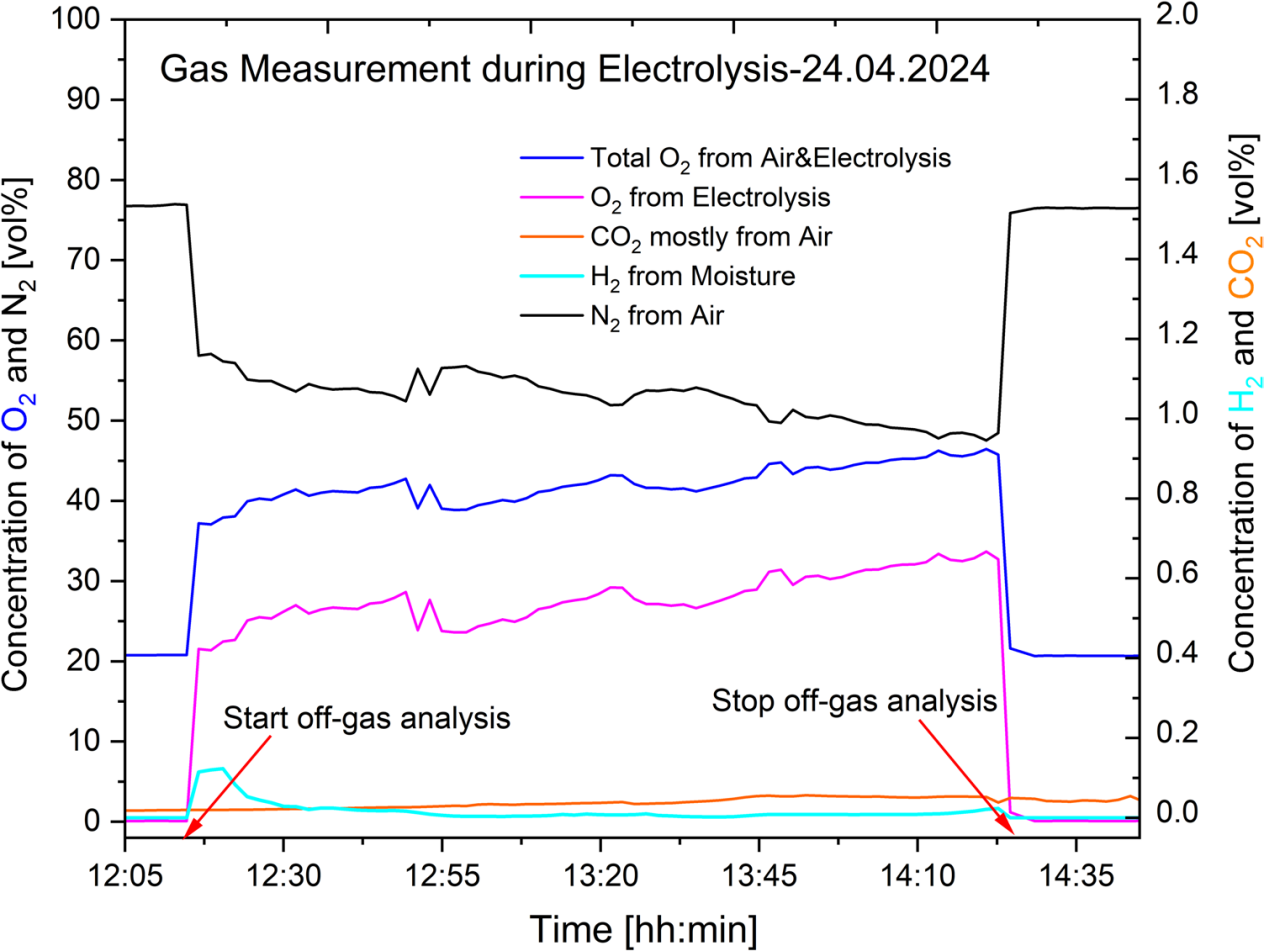
Off-gases from the REVEAL electrolysis process measured on 24.04.2024

**Fig. 9:**
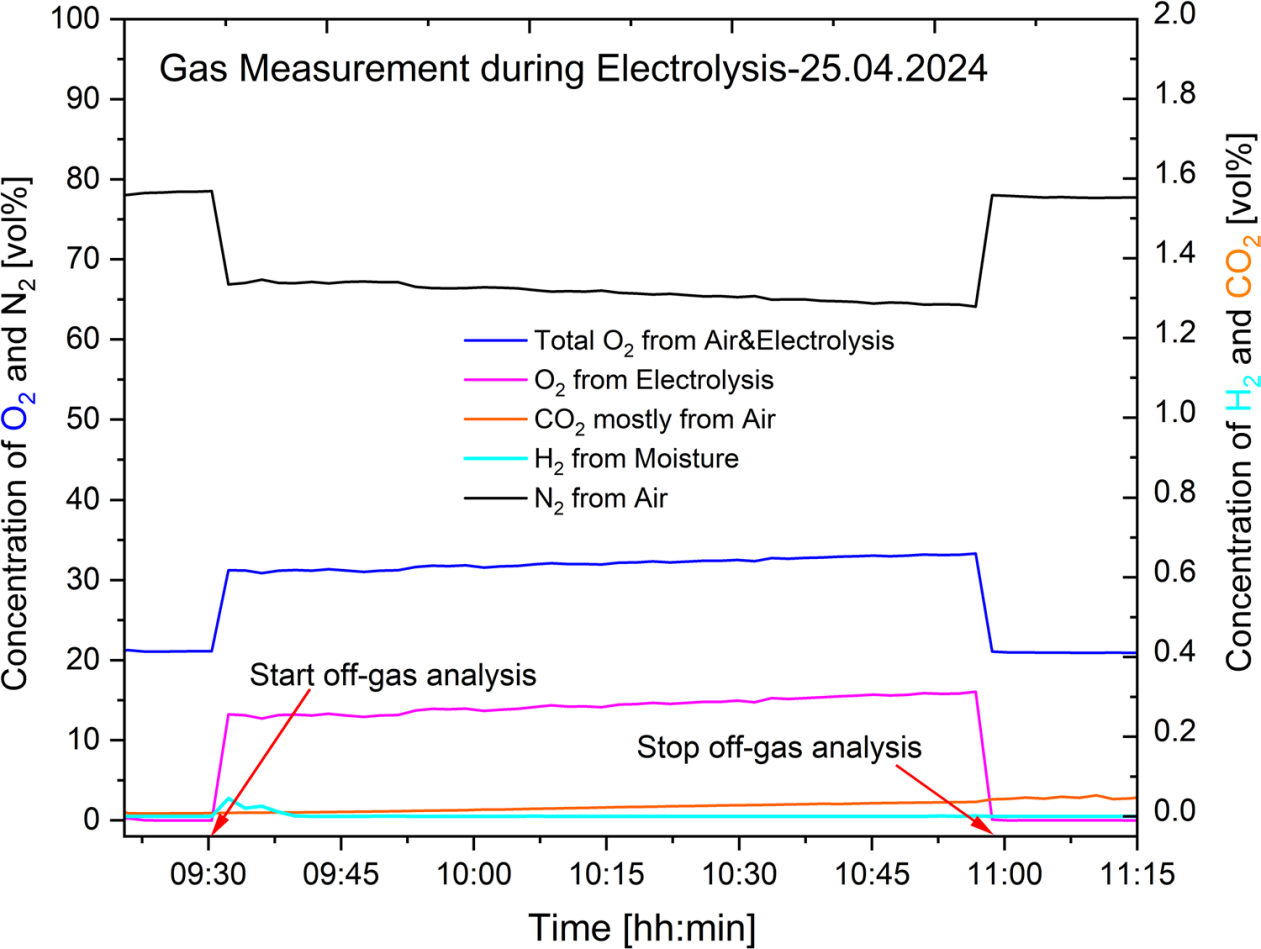
Off-gases from the REVEAL electrolysis process that was measured on 25.04.2024

The first off-gas characterization activity done in April 2024 showed the presence of oxygen, hydrogen, nitrogen and carbon dioxide using an Agilent 490-PRO Micro-GC equipped with a TCD as initially mentioned. The results are presented in Figs. 7, 8, and 9. For the O_2_ concentrations, the measurement instrument analysed the total amount present, which included the contribution from air. Thus, calculations based on Eqs. (16) and (17) were done to identify the right amount of oxygen resulting from the electrolysis process.

16 $$ {\mathrm{Conc}}{.}\;{\mathrm{of}}\;{\mathrm{O}}_{2} \;{\mathrm{from}}\; {\mathrm{air}}\;{\mathrm{mixed}}\;{\mathrm{with}}\;{\text{ off - gas}}\; = \;\frac{{{\mathrm{Volume}}\;{\mathrm{ratio}}\;{\mathrm{of}}\;{\mathrm{O}}_{2} \;{\mathrm{in}}\;{\mathrm{air}}}}{{{\mathrm{Volume}}\;{\mathrm{ratio}}\;{\mathrm{of}}\;{\mathrm{N}}_{2} \;{\text{ in}}\;{\mathrm{air}}}}\; \times \;{\mathrm{measured}}\;{\mathrm{conc}}{.}\; {\mathrm{of}}\;{\mathrm{N}}_{2} $$

17 $$ {\mathrm{Conc}}{.} \;{\mathrm{of}}\;{\mathrm{O}}_{2} \;{\mathrm{from}}\;{\mathrm{electrolysis}}\;{\mathrm{process}}\; = \;{\mathrm{measured}}\;{\mathrm{conc}}{.}\;{\mathrm{of}}\;{\mathrm{O}}_{2} \; - \;{\mathrm{calculated}}\;{\mathrm{conc}}{.}\;{\mathrm{of}}\;{\mathrm{O}}_{2} $$

18 $$ {\mathrm{Total}}\; {\mathrm{conc}}{.}\;{\text{ of}}\;{\mathrm{O}}_{2} \;{\mathrm{from}}\;{\text{ air}}\;{\mathrm{and}}\; {\mathrm{electrolysis}}\;{\mathrm{process}}\; = \;{\mathrm{measured}}\;{\mathrm{conc}}{.}\;{\text{ of}}\;{\mathrm{O}}_{2} $$

The data gathered during the November 2024 measurements included HF, which is expected to be present due to contact between the fluoride melt and moisture from the alumina or surrounding air.

**Fig. 10:**
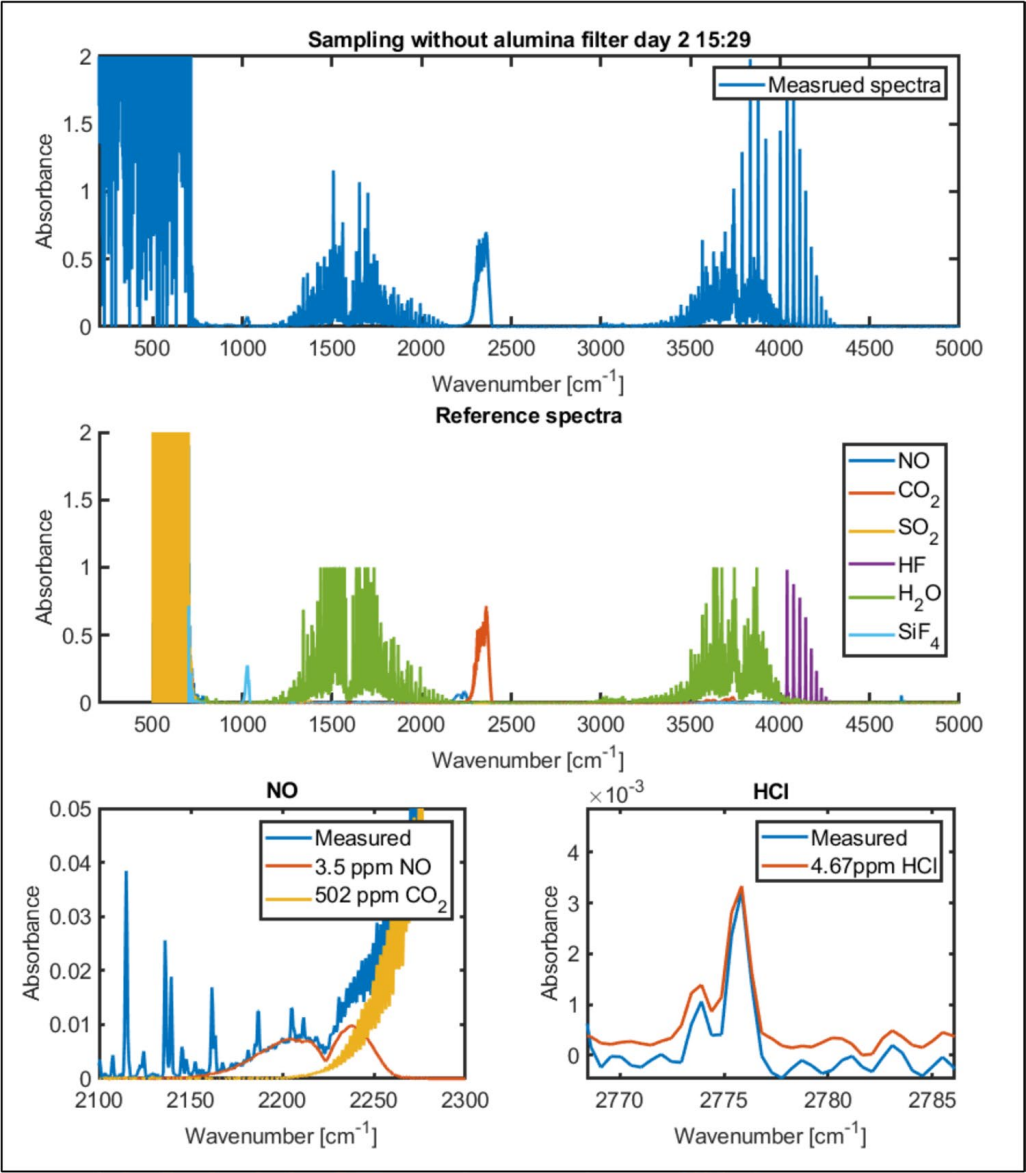
Identification process of FTIR data. The measured spectra (top) are compared to reference spectra (middle), where the most prominent components are found. components with minor contributions are then identified

The measurements were conducted as burst measurements for 20–60 min which was the time allowed before the filters were packed with bath vapours and had to be cleaned. As the operation of the electrolysis process is continuous except the tapping there are no expected short-term variations in the off-gas concentration (although longer term variations could be a result of cell degradation). To increase analytical precision averages of the measurements are used in signal treatment. The identification process for FTIR data is illustrated in Fig. 10. The main components are first found. Then components with smaller contributions is identified. In the lower-left corner, we can clearly see that the measured signal (blue) has an increase before the onset of the CO_2_ peak (yellow). This fits well with the signature of a small NO contribution of about 3.5 ppm. Likewise, we can see a small signal from HCl between 2700 cm^−1^ and 2900 cm^−1^.

**Fig. 11:**
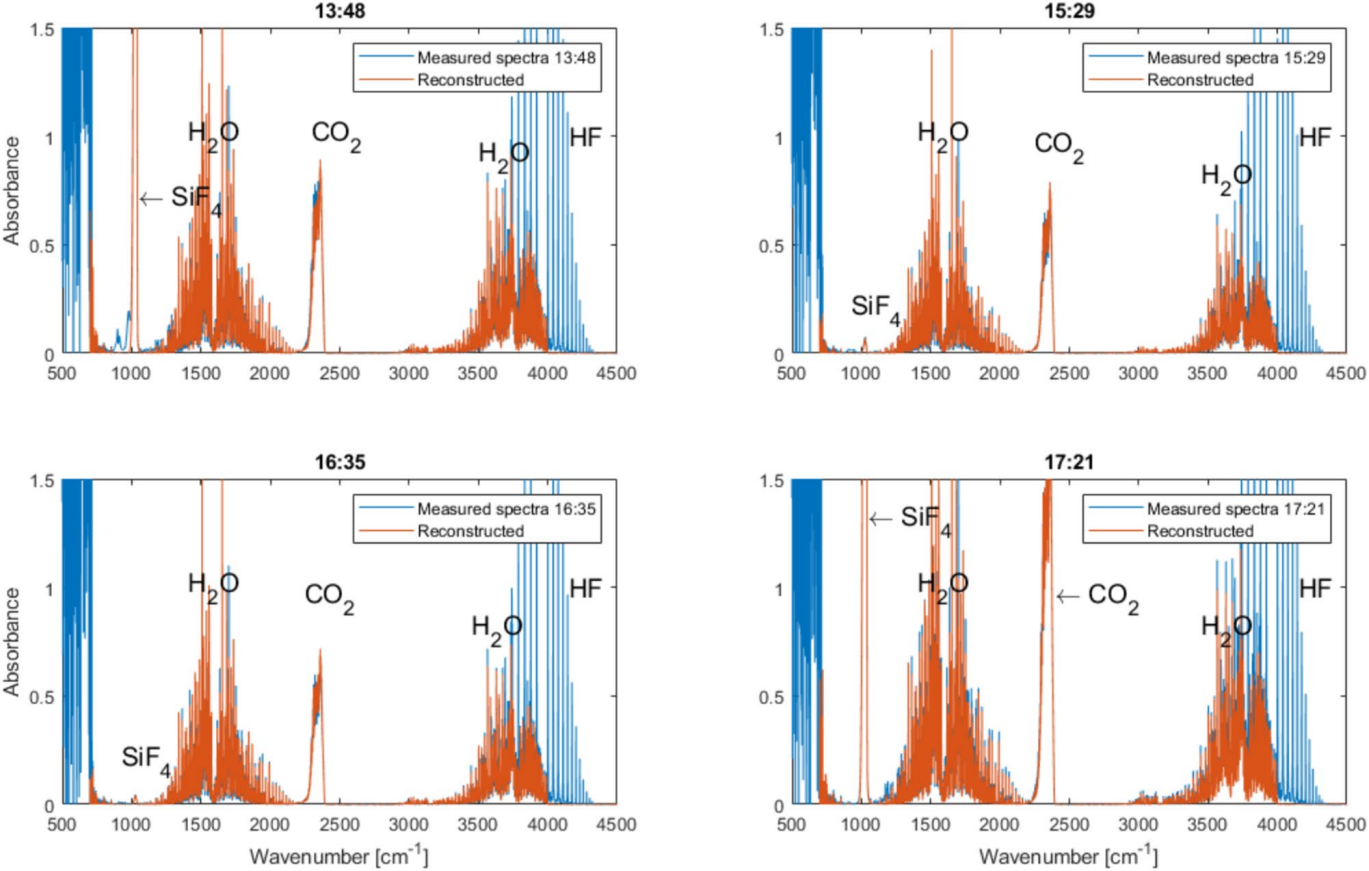
Plots for the fitting of average concentrations for the 4 measurements on the 5th of November, showing original measured data in blue and data fitted from reference spectra in orange. HF was not possible to fit due to high concentrations (Color figure online)

Figure 11 illustrates plots from the fitting of average concentrations for the 4 measurements taken. It shows the original measured data in blue and data fitted from reference spectra in orange. HF was not possible to fit due to the extremely high concentrations measured.

**Fig. 12:**
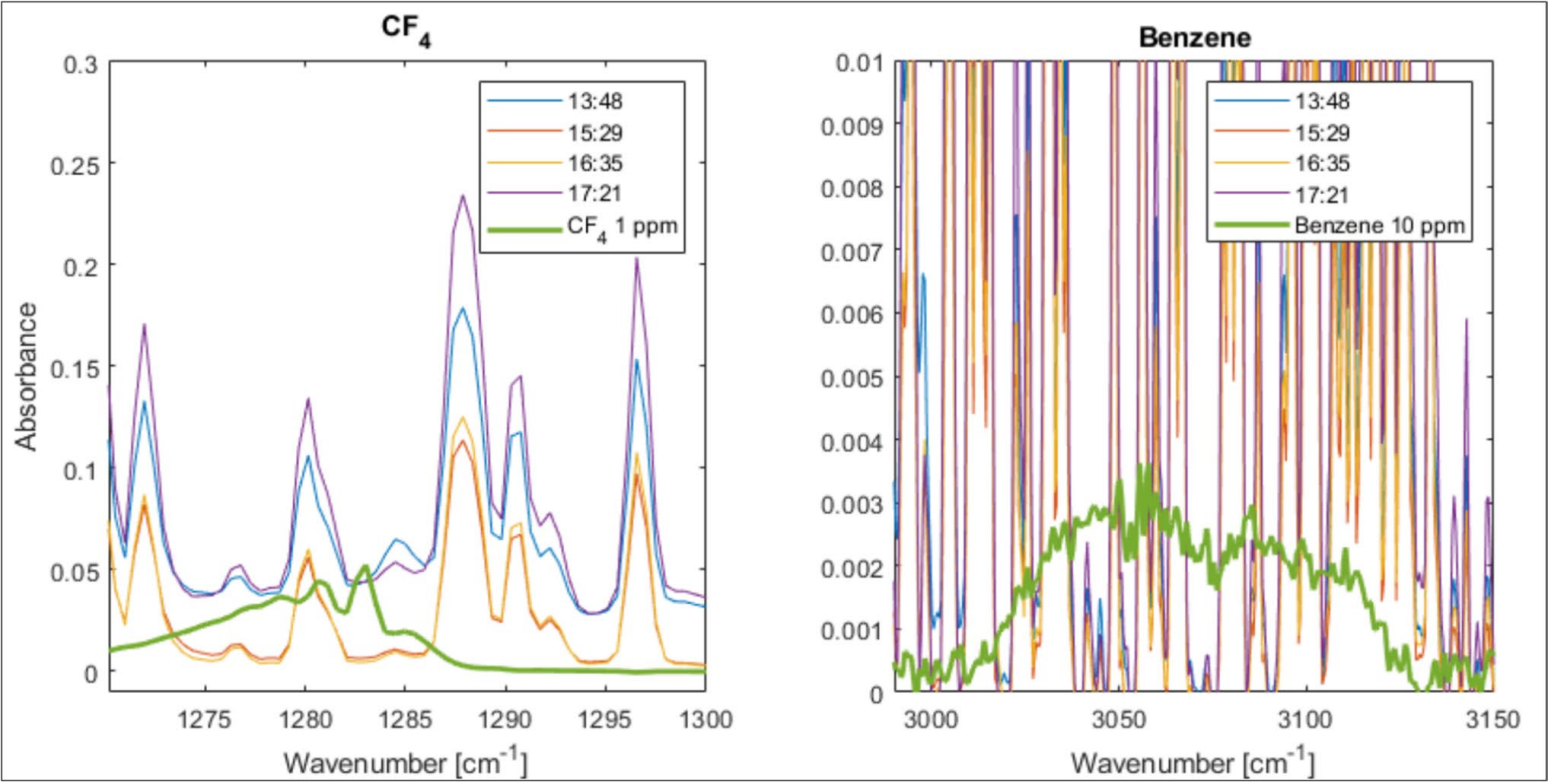
Positive confirmation that CF_4_ and benzene are not present in the off-gas

To be sure that there were none of the unwanted carbon gases, such as CF_4_ and aromatic hydrocarbons, present, they were specifically sought for, even though they should not be expected in the gas mixture. From the plots in Fig. 12, we can, therefore, see that neither CF_4_ nor Benzene exist in any of the off-gas measurements below the calibration limits of 1 and 10 ppm, respectively. For CF_4,_ there is a peak at 1283 cm^−1^ that is not reflected in any of the measured data. For benzene, there is a broad peak from 3000 to 3130 cm^−1^, while all measurement data show just a narrow overlaying water with signals contacting the baseline throughout the wavelength region.

HF can be qualitatively identified by FTIR, but the concentration is so high that it is way outside the highest quantification limit of this analyser. HF was, therefore, quantified by TD-laser. The high levels of HF, typically from 10,000–15,000 ppm, fit well with what can be observed from aluminum smelters, where there is 1% CO_2_ (produced gas) and 500–1000 ppm HF. Since the HF evolution is directly linked to the moisture content of the raw materials, Al_2_O_3_, its flow into the process will result in a more concentrated off-gas proportionally to the heightened levels of HF. With a typical O_2_ concentration from electrolysis of 20–40% we would expect to see at least these levels of HF in the off-gas, or at least 20–40 times higher than in off-gas from Hall–Héroult cells. In Table 1, the values of the estimated gas concentrations are summarised for analysis from both the FTIR and the TDL (HF).

**Table 1:** Estimated gas concentrations for the four selected timeframes

	13:48	15:29	16:35	17:21
HF (ppm)	10,481	12,992	9785	10,755
CO_2_ (ppm)	623	553	502	1335
SiF_4_ (ppm)	488	3.75	2.25	946
NO (ppm)	3.5	3.5	3	3
SO_2_ (ppm)	10		–	
H_2_O (%)	0.926	0.732	0.793	1.22
HCl (ppm)	–	4.67	–	4.67

## Conclusions and Discussion

**Fig. 13:**
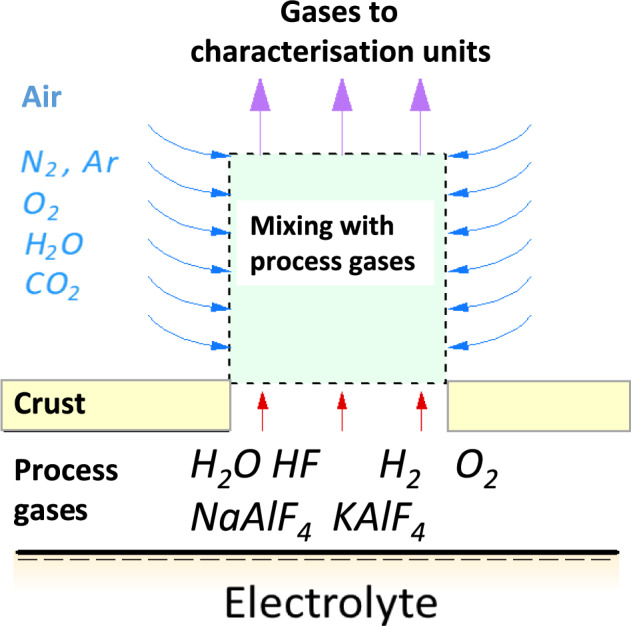
Mixing of air with process gas (redrawn from Senanu et al. [9])

N_2_ and a portion of the CO_2_, H_2_O and O_2_ detected by the characterization units are assumed to result from air mixing with the process gases as the electrolysis setup was not completely closed. A sketch illustrating the mixing of process gases with air entering the electrolysis setup is given in Fig. 13.

A confirmation that air mixes with the process gases is visible from the gas measurement data given in Figs. 7, 8 and 9. A GC equipped with TCD was used to obtain the data presented. In these figures, the levels of oxygen, O_2_, and nitrogen, N_2_, before connecting the off-gas measurement unit to the electrolysis cell are very similar to the levels in air. The concentration of O_2_, as expected, increases once the gas collection unit is connected to the electrolysis cell. The concentration of N_2,_ on the other hand, decreases after the connection. As there is no source of nitrogen, the N_2_ measured can only come from air, thereby confirming that the process gas is mixed with air. Oxygen concentration increases, whereas nitrogen concentration decreases whenever the collection unit is connected, and then both go back to levels similar to their concentrations in air once the unit is disconnected. The H_2_ gas observed is assumed to form by reactions between moisture and the dissolved aluminum. The increased concentration of H_2_ when the collection unit is connected is assumed to result from the relatively high amount of moisture introduced. The HF scrubber of the characterization unit absorbs moisture from the atmosphere when it is not connected. Upon connection, this moisture is introduced to the electrolyte containing dissolved aluminum. The small amount of CO_2_ observed is also from air mixing, since there is no source of carbon in the system. The seemingly gradual increase of CO_2_ is attributed to the absorbed CO_2_ by the HF scrubber before the connection of the collection unit. The gradual increase is due to the release of the absorbed CO_2_ during the measurement.

Results from the second campaign that utilised an FTIR unit are illustrated in Figs. 10, 11 and 12. Figure 11 suggests the presence of HF, SiF_4_, CO_2_, and H_2_O in addition to O_2,_ which is the main process gas, as shown by the previous data. H_2_O can also be assumed to result from air mixing and moisture from the alumina addition, which confirms the formation of HF. There were periods of very high HF concentrations during the alumina additions as can be seen in Fig. 11. Minor concentrations of HCl and NO were also detected. HCl might originate from impurities in the alumina, whereas NO could result from the presence of N_2_ from the air.

The off-gas characterization campaigns conclude that the main off-gas generated during the inert electrode electrolysis process is O_2_, with HF being the 2nd most abundant, H_2_ is also formed in small quantities, whereas SiF_4_ is present in most of the analysed data but has a significantly larger variation than any of the other gas components. The presence of CO_2_ and N_2_ was assumed to result mainly from air mixing.
